# Supplementary material for: Appropriate Reference Genes for RT-qPCR Normalization in Various Organs of Anemone flaccida Fr. Schmidt at Different Growing Stages
Source: Genes (Basel). 2021 Mar 23;12(3):459. doi: 10.3390/genes12030459 (PMC8005022; doi:10.3390/genes12030459)
Supplement: Supplementary file 1 [file genes-12-00459-s001.pdf]

genes MDPI

Supporting Information

Table S1 Ranking of 10 candidate reference genes by geNorm (G), NormFinder (N) and Bestkeeper (B) methods. Total: all rhizome and leaf samples of cultivated and wild plants; SR: rhizomes at seeding stage; SL: leaves at seeding stage; FR: rhizomes at flowering stage; FL: leaves at flowering stage; WR: rhizomes at withering stage.

Sample	Software	1	2	3	4	5	6	7	8	9	10
Total	G	*PUBQ*	*ETIF1a*	*EF1A*	*hh2a*	*α-tubulin*	*ubiquitin*	*PKII*	*GAPDH*	*β-tubulin*	*28SrRNA*
	N	*PUBQ*	*hh2a*	*ETIF1a*	*EF1A*	*ubiquitin*	*PKII*	*α-tubulin*	*β-tubulin*	*GAPDH*	*28SrRNA*
	B	*PUBQ*	*ETIF1a*	*β-tubulin*	*hh2a*	*EF1A*	*ubiquitin*	*GAPDH*	*PKII*	*28SrRNA*	*α-tubulin*
SR	G	*PUBQ*	*ETIF1a*	*EF1A*	*α-tubulin*	*ubiquitin*	*hh2a*	*28SrRNA*	*PKII*	*GAPDH*	*β-tubulin*
	N	*EF1A*	*PUBQ*	*ETIF1a*	*hh2a*	*α-tubulin*	*ubiquitin*	*PKII*	*28SrRNA*	*β-tubulin*	*GAPDH*
	B	*PUBQ*	*EF1A*	*ETIF1a*	*β-tubulin*	*tubulin*	*ubiquitin*	*hh2a*	*GAPDH*	*28srRNA*	*PKII*
SL	G	*α-tubulin*	*ETIF1a*	*EF1A*	*PUBQ*	*hh2a*	*ubiquitin*	*PKII*	*GAPDH*	*β-tubulin*	*28SrRNA*
	N	*PUBQ*	*ETIF1a*	*α-tubulin*	*hh2a*	*EF1A*	*ubiquitin*	*GAPDH*	*PKII*	*β-tubulin*	*28SrRNA*
	B	*PUBQ*	*hh2a*	*ETIF1a*	*β-tubulin*	*28SrRNA*	*EF1A*	*α-tubulin*	*PKII*	*GAPDH*	*ubiquitin*
FR	G	*EF1A*	*ETIF1a*	*PUBQ*	*α-tubulin*	*hh2a*	*ubiquitin*	*28SrRNA*	*PKII*	*β-tubulin*	*GAPDH*
	N	*PUBQ*	*ETIF1a*	*α-tubulin*	*EF1A*	*ubiquitin*	*hh2a*	*PKII*	*β-tubulin*	*GAPDH*	*28SrRNA*
	B	*ETIF1a*	*PUBQ*	*EF1A*	*ubiquitin*	*28SrRNA*	*β-tubulin*	*hh2a*	*GAPDH*	*PKII*	*α-tubulin*
FL	G	*α-tubulin*	*ETIF1a*	*EF1A*	*PUBQ*	*hh2a*	*GAPDH*	*β-tubulin*	*ubiquitin*	*28SrRNA*	*PKII*
	N	*PUBQ*	*ETIF1a*	*EF1A*	*hh2a*	*ubiquitin*	*PKII*	*28SrRNA*	*α-tubulin*	*β-tubulin*	*GAPDH*
	B	*ETIF1a*	*PUBQ*	*β-tubulin*	*EF1A*	*PKII*	*ubiquitin*	*28SrRNA*	*α-tubulin*	*hh2a*	*GAPDH*
WR	G	*EF1A*	*ETIF1a*	*PUBQ*	*hh2a*	*α-tubulin*	*ubiquitin*	*28SrRNA*	*PKII*	*GAPDH*	*β-tubulin*
	N	*PUBQ*	*EF1A*	*ETIF1a*	*hh2a*	*α-tubulin*	*GAPDH*	*PKII*	*ubiquitin*	*28SrRNA*	*β-tubulin*
	B	*ETIF1a*	*PUBQ*	*EF1A*	*α-tubulin*	*β-tubulin*	*GAPDH*	*ubiquitin*	*hh2a*	*28SrRNA*	*PKII*

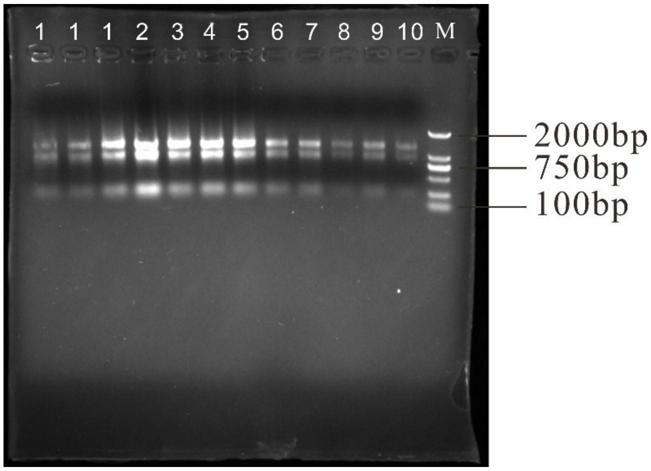

Fig. S1 Agarose gel electrophoresis of RNA in different samples

1: cultivated rhizomes at seeding stage (CSR); 2: wild rhizomes at seeding stage (WSR); 3: cultivated leaves at seeding stage (CSL); 4: wild leaves at seeding stage (WSL); 5: cultivated rhizomes at flowering stage (CFR); 6: wild rhizomes at flowering stage (WFR); 7: cultivated leaves at flowering stage (CFL); 8: wild leaves at flowering stage (WFL); 9: cultivated rhizome at withering stage (CWR); 10: wild rhizomes at withering stage (WWR); M: Marker DL2000.

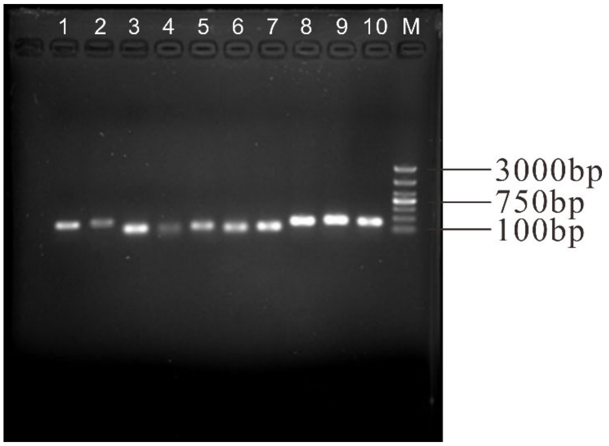

Fig. S2 qPCR amplification specificity of 10 candidate reference genes. Amplification fragments were separated by 2% agarose gel electrophoresis.

1: *GAPDH*; 2:*β-tubulin*; 3: *EF1A*; 4: *28SrRNA*; 5: *ETIF1a*; 6: *hh2a*; 7: *Ubiquitin*; 8: *PKII*; 9:*α-tubulin*; 10: *PUBQ*; M: DNA marker.

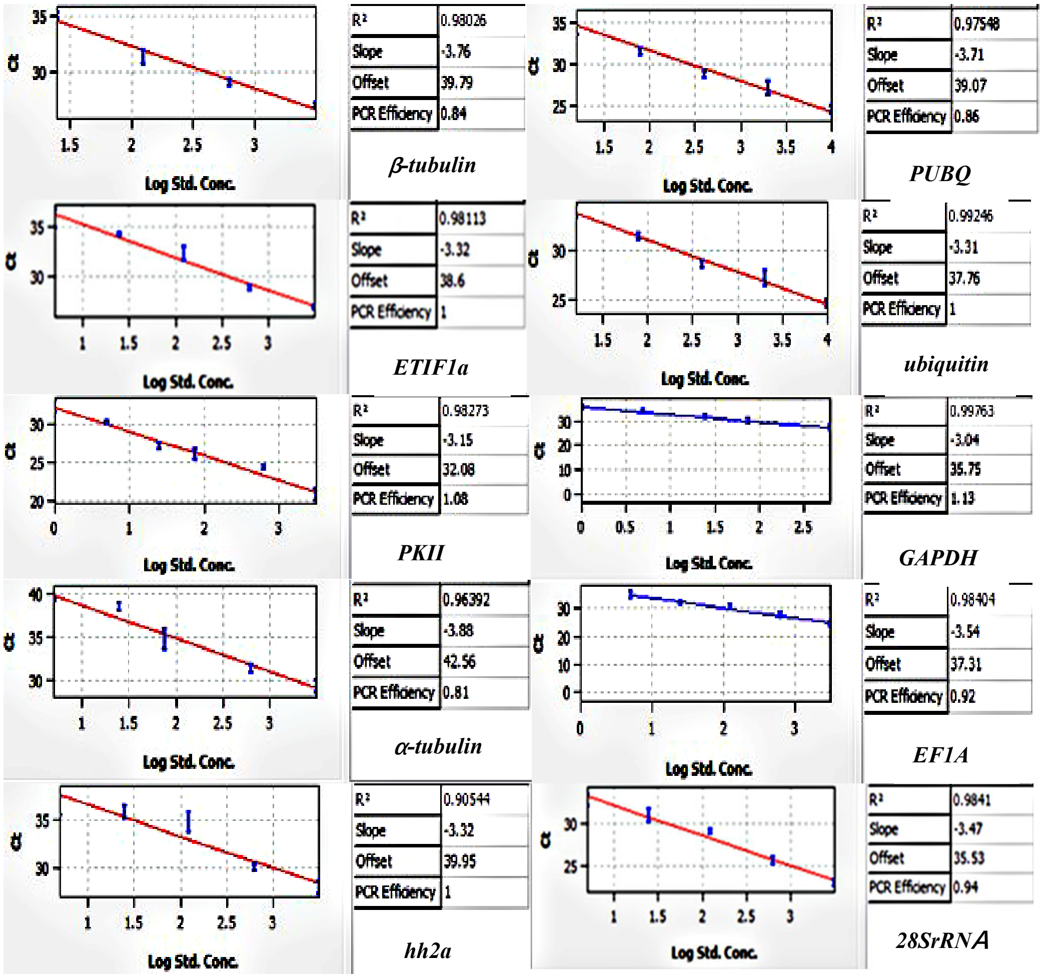

Fig. S3 Amplification efficiencies of 10 candidate reference genes
